# Management of Low-Grade Appendiceal Mucinous Neoplasm with Extensive Peritoneal Spread Diagnosed during Pregnancy: Two Case Reports and Literature Review

**DOI:** 10.1155/2020/8853704

**Published:** 2020-10-15

**Authors:** Ekaterina Baron, Vadim Gushchin, Mary Caitlin King, Andrei Nikiforchin, Armando Sardi

**Affiliations:** Department of Surgical Oncology, The Institute for Cancer Care at Mercy, Mercy Medical Center, 227 St. Paul Place, 4th Floor Weinberg, Baltimore, Maryland 21202-2001, USA

## Abstract

**Background:**

Clinical decisions in patients with peritoneal dissemination of low-grade appendiceal mucinous neoplasms (LAMN) diagnosed during pregnancy are challenging. However, their slow progression and favorable prognosis allow deferring definitive treatment until after spontaneous delivery, a reasonable period of breastfeeding, and fertility preservation. *Case Presentation*. Two pregnant patients were incidentally diagnosed with LAMN and extensive peritoneal spread at 20 weeks gestation and at cesarean section. Treatment with cytoreductive surgery and hyperthermic intraperitoneal chemotherapy in both cases was delayed until spontaneous delivery at term and breastfeeding in the first patient and breastfeeding and fertility preservation in the second patient. Both patients remain disease-free for over 5 years, and their children are healthy. The literature review highlights the challenges that physicians face in treating pregnant patients with stage IV appendiceal tumors.

**Conclusion:**

Pregnancy management decisions in patients with peritoneal spread from mucinous appendiceal tumor should be based on understanding the tumor biology and prognosis. Definitive treatment in pregnant patients with favorable tumors, such as LAMN, may be delayed until spontaneous delivery without compromising maternal survival.

## 1. Introduction

Treating neoplasms diagnosed during pregnancy entails weighing the risks and benefits for both mother and fetus. This requires considering multiple factors, such as tumor biology and prognosis, stage, gestational age at diagnosis, efficacy, toxicity, invasiveness of required treatment, and patient preferences. Particularly, the management of stage IV tumors with extensive dissemination during pregnancy poses additional challenges because it demands rapid and complex ethical decisions, which should be based on a deep understanding of tumor behavior and all available options.

Appendiceal mucinous neoplasms (AMN) are a rare group of malignancies with high heterogeneity of histopathologic subtypes and survival outcomes [1]. They range from low-grade neoplasms with favorable prognosis to high-grade tumors with significantly worse outcomes. At the same time, up to 20% of AMN present with peritoneal carcinomatosis (PC) [2], considered stage IV according to the American Joint Committee on Cancer (AJCC) 8^th^ edition [3]. However, excellent long-term outcomes can be achieved in AMN with cytoreductive surgery and hyperthermic intraperitoneal chemotherapy (CRS/HIPEC) [4].

Diagnosis of PC during pregnancy is often considered to be a life-threatening condition where oncological concerns of the mother outweigh fetal risks. In the few published cases of PC from low-grade appendiceal tumors diagnosed during pregnancy, all managed with early labor induction, early cesarean section, or pregnancy termination in favor of faster definitive treatment [5–8]. However, the favorable prognosis and slow progression of LAMN, even with extensive PC, allows performing CRS/HIPEC after spontaneous delivery, which minimizes fetal risks without compromising maternal survival [9] We present two unique cases of LAMN with PC diagnosed during pregnancy and managed with delayed CRS/HIPEC in the postpartum period accompanied by a literature review.

## 2. Case #1

**Figure 1:**
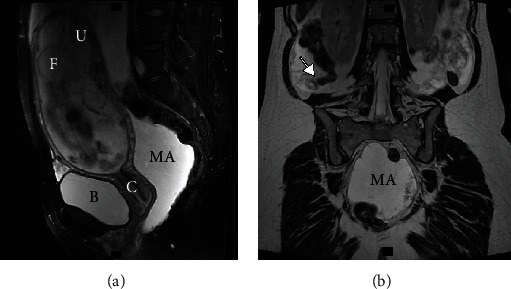
(a) Abdominal and pelvic MRI (sagittal plane) without IV contrast shows a gravid uterus and a substantial amount of T2 hyperintense fluid. (b) Abdominal and pelvic MRI (coronal plane) without IV contrast demonstrates an enlarged perforated appendix with extraluminal mucin (arrow) and T2 hyperintense fluid collection in the pelvis. B: bladder; C: cervix; F: fetus; IV: intravenous; MA: mucinous ascites; MRI: magnetic resonance imaging; U: uterus.

**Figure 2:**
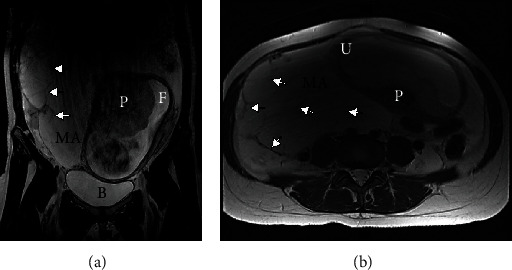
(a), (b) Abdominal and pelvic MRI without IV contrast ((a) coronal plane, (b) axial plane) shows a gravid uterus and significant amount of T2 hyperintense fluid, some of which is loculated with internal septations (arrows). B: bladder; F: fetus; IV: intravenous; MA: mucinous ascites; MRI: magnetic resonance imaging; P: placenta; U: uterus.

A 31-year-old woman, gravida 2 para 1, with a history of asthma and anemia presented with a large intra-abdominal mass and ascites found incidentally on routine prenatal ultrasound (US) at 20 weeks gestation. Prior to that, the patient had an uneventful pregnancy and skipped US during the first trimester prenatal screening. An ovarian primary tumor was suspected and abdominopelvic magnetic resonance imaging (MRI) without contrast was performed within one week. MRI showed a disrupted appendiceal tip surrounded by soft tissues and fluid, as well as a substantial amount of abdominopelvic fluid with internal septations and debris, suggesting an appendiceal mucinous neoplasm with PC (Figures 1(a), 1(b), 2(a), 2(b)). Appendectomy, right salpingo-oophorectomy, omentectomy, and peritoneal biopsy were performed at 21 weeks gestation and pathology confirmed LAMN with cellular mucinous peritoneal implants (AJCC 8^th^ stage IVA).

The patient was referred to a peritoneal surface malignancy center. Considering the favorable prognosis of LAMN even with extensive peritoneal spread, we assessed the risk of rapid tumor progression as low and recommended to defer definitive treatment until the postpartum period. A healthy 2,950 g (6.6 lbs.) male was born by uncomplicated spontaneous vaginal delivery at 38 weeks gestation. Staging with computed tomography (CT) of the chest, abdomen, and pelvis revealed multiple bilateral subdiaphragmatic, omental, mesenteric, and liver capsule mucinous implants and no extraperitoneal metastases. Tumor markers (CEA, CA 125, and CA 19-9) were normal. After 4 months of breastfeeding, the patient underwent CRS/HIPEC with 40 mg mitomycin-C heated to 41-42°C for 90 minutes using the closed technique. The peritoneal cancer index (PCI) (range 0-39) [10] was 37. The surgery lasted 708 minutes with an estimated blood loss of 1,100 ml. Complete cytoreduction with residual small membranes on the small bowel (completeness of cytoreduction (CC) score 1) [4] was achieved. The postoperative period was complicated by anemia requiring red blood cell transfusion. The patient was discharged on postoperative day (POD) 9 with deep vein thrombosis (DVT) prophylaxis (40 mg of enoxaparin daily). However, she was readmitted twice: first with splenic and portal vein thrombosis (POD 15) and then with rectal bleeding (POD 30) after subsequent increase in anticoagulant dose. Regular follow-up included physical examinations; imaging of the chest, abdomen, and pelvis; and tumor markers every 6 months. At 63 months of follow-up, the patient remains disease-free and her child is healthy.

## 3. Case #2

A 31-year-old healthy woman, gravida 3 para 0, underwent cesarean section at 40 weeks gestation due to premature rupture of membranes and protracted labor and delivered a healthy 3,561 g (7.9 lbs.) male newborn. The patient had a history of lower back pain and mild anemia throughout the pregnancy and started experiencing diarrhea at 35 weeks. The full prenatal screening, including US in each trimester, revealed only an echogenic intracardiac focus of the fetus. Otherwise, the pregnancy was uneventful. During cesarean section, mucin originating from the appendix tip was found adherent to the uterus, right ovary, and fallopian tube. Appendectomy and peritoneal biopsy were performed, and pathology showed LAMN with PC (AJCC 8^th^ stage IVA).

The chest, abdominal, and pelvic CT scan 3 weeks after delivery showed perihepatic and perisplenic capsular implants. Tumor marker assessment showed elevated CEA up to 8.8 ng/ml (N 0-5 ng/ml) and normal CA 125 and CA 19-9. Definitive treatment was delayed for breastfeeding and oocyte retrieval for cryopreservation of 4 embryos. Three months postdelivery, the patient underwent CRS/HIPEC with 90-minute perfusion of 40 mg mitomycin-C heated to 41-42°C. PCI was 27, and a complete cytoreduction was achieved (CC-score 0) after 487 minutes of surgery. The patient was discharged on POD 8 without major complications. She was readmitted several times over the last 5 years with small bowel obstructions that resolved nonoperatively. Three years after CRS/HIPEC, the patient had a second child via surrogate maternity using a frozen embryo. The follow-up was the same as the previous case. After 67 months, the patient is disease-free and her children are doing well.

## 4. Discussion

The presented patients were diagnosed with a rare appendiceal tumor and PC at an advanced gestational age of pregnancy: 20 weeks and 40 weeks during cesarean section. To our knowledge, these are the first published cases of peritoneal spread from LAMN diagnosed during pregnancy managed with delayed CRS/HIPEC, which, for patient #1, allowed spontaneous delivery of a healthy baby at 38 weeks with 4 months of breastfeeding and, for patient #2, 3 months of breastfeeding and cryopreservation of embryos that were eventually used successfully.

Diagnosis of PC at any stage of pregnancy is dramatic and associated with multiple challenges for both physician and patient. While there are numerous reports of appendiceal tumors diagnosed during pregnancy (Tables 1 and 2), we found only 6 with PC: 4 diagnosed during pregnancy and 2 of diagnosed during cesarean section. Of the four cases diagnosed during pregnancy, one was managed with preterm induction of labor at 35 weeks, two were managed with an early cesarean section at 30 and 33 weeks, and one was terminated at 18 weeks [5–8]. The time of definitive treatment in the two cases diagnosed at cesarean section is unknown as patients were referred to outside facilities [10, 11]. This may demonstrate that pregnancy termination or early labor induction with rushing CRS/HIPEC is a common strategy among peritoneal malignancy centers to treat patients with a peritoneal spread from AMN. In our center, we opted to delay CRS/HIPEC and allow the pregnancy to progress naturally.

The approach to delay definitive treatment in our cases was based on the well-known favorable prognosis of LAMN with extensive peritoneal spread treated with CRS/HIPEC. Two previously published cases of well-differentiated appendiceal adenocarcinoma with PC, which currently is considered to be the same grade (G1) and prognostic stage (IVA) as LAMN, managed their patients with early labor induction (35 weeks) and early cesarean section (33 weeks) followed by CRS/HIPEC in 2.5 weeks and 2 months, respectively [3, 5, 7]. Moreover, Haase et al. proposed managing peritoneal surface malignancies diagnosed during pregnancies with early induction and delaying treatment to 35 weeks if diagnosed in the 2^nd^ trimester [5]. However, labor at 34-37 weeks of gestation refers to late preterm delivery and is associated with increased risk for numerous complications for the infant and should be avoided when possible [11]. We believe that the histopathology and disease pathogenesis must be considered carefully before inducing early delivery or termination. In case #1, the diagnosis was made in the 2^nd^ trimester (20 weeks) during routine prenatal screening. Previous studies demonstrate an excellent prognosis of LAMN patients with 5-year overall survival up to 80-96% 9, 12]. This data suggests that the slow progression of LAMN allows for a natural progression of pregnancy and deferring CRS/HIPEC for a reasonable period. Therefore, we opted to postpone CRS/HIPEC for 8 months in case #1 until spontaneous at term delivery with subsequent breastfeeding and for 4 months in case #2 for breastfeeding and fertility preservation without compromising survival outcomes in either case as it is oncologically safe.

Diagnosing and staging AMN might be challenging during pregnancy due to the limited availability of safe diagnostic methods and nonspecific clinical presentation. Considering patient and fetus risks, only tests that may influence clinical management should be performed. Once peritoneal spread or local appendiceal tumor is suspected during prenatal pelvic US, additional imaging is required for staging and making decisions regarding diagnostic surgery. Abdominopelvic MRI without contrast is a safe and informative alternative to CT during pregnancy for clarifying the diagnosis of peritoneal lesions [13, 14]. The MRI in case #1 demonstrated a disrupted appendiceal tip and signs of mucin distributed throughout the abdomen and pelvis (Figures 1 and 2). These findings shifted our focus from initially suspected ovarian origin to appendiceal tumor and directed the diagnostic surgery.

Defining histopathologic subtype and stage is crucial for establishing treatment in patients with PC. We believe that diagnostic open or laparoscopic surgery for appendectomy, peritoneal biopsy, and thorough revision of the abdomen and pelvis is reasonable and should be performed in all patients with PC on imaging regardless of pregnancy stage. Laparoscopy has advantages in this case as it allows inspecting the majority of the abdominal cavity, including the upper abdomen. However, the use of laparoscopy in pregnant women is controversial. A meta-analysis of 11 retrospective studies showed that laparoscopic appendectomy is associated with higher fetal loss in pregnant women compared to open surgery [15]. At the same time, the largest study of almost 20,000 pregnant women reported a higher risk of adverse obstetrical events including miscarriages, preterm labor, and intrauterine death after open surgery [16]. In case #1, open appendectomy and peritoneal biopsy were performed, rather than full abdominal cavity exploration, for further histopathology assessment since PC had been already confirmed with MRI.

Regardless of the approach, surgical specimens obtained from the diagnostic surgery require meticulous pathologic assessment. Frozen sections should not be used to diagnose appendiceal tumors due to their complex pathology and low concordance between frozen sections and final pathology [17]. Fine needle biopsy of appendiceal neoplasm should also be avoided due to potential peritoneal spread and inadequate sampling [18]. Given the rarity and complexity of appendiceal neoplasm histopathology, we recommend the removal of the entire appendix and revision of slides at a specialized peritoneal surface malignancy center as it can drastically affect the management [19]. In both presented cases, pathology assessment of specimens, including the entire primary appendiceal tumor and peritoneal biopsy, confirmed LAMN with peritoneal spread which directed our delayed treatment approach.

**Figure 3:**
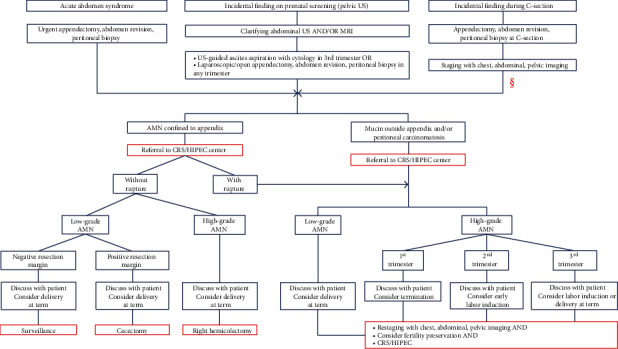
Algorithm of appendiceal mucinous neoplasm management diagnosed during pregnancy. ^§^The algorithm here is consistent with that for the first two scenarios except pregnancy management. AMN: mucinous appendiceal neoplasm; CRS/HIPEC: cytoreductive surgery with hyperthermic intraperitoneal chemotherapy; C-section: cesarean section; MRI: magnetic resonance imaging; PC: peritoneal carcinomatosis; US: ultrasound.

AMN management depends on histopathologic subtype, prognosis, and stage at presentation. Based on previously published and our cases, appendiceal tumors during pregnancy usually present as one of the three clinical scenarios: (1) local tumor with/without appendix rupture with clinical signs of acute abdomen (*n* = 13 cases), (2) symptomatic or asymptomatic PC diagnosed incidentally during prenatal screening (*n* = 5 cases), and (3) AMN with/without PC incidentally found on cesarean section (*n* = 9 cases) (Tables 1 and 2). Each of these scenarios requires a different strategy to determine the optimal treatment plan and make decisions regarding pregnancy preservation and timing of definitive treatment (Figure 3). The treatment of tumor confined to the appendix usually requires only local resection varying from appendectomy/cecectomy for low-grade neoplasms to right hemicolectomy in high-grade tumors. Pregnant patients with ruptured appendiceal lesions and/or peritoneal involvement require CRS/HIPEC and should be referred to a specialized peritoneal surface malignancy center as soon as possible. Considering the various prognoses of AMN histopathologic subtypes, successful treatment does not always require pregnancy termination or early labor induction. Therefore, decisions regarding pregnancy management and preservation ought to be made case by case at a specialized peritoneal surface malignancy center by a multidisciplinary team including a CRS/HIPEC surgeon and gynecologist.

Thrombotic complications after CRS/HIPEC performed in the postpartum period might be a specific hazard. In case #1, the patient developed a rare and threatening complication of the portal and splenic vein thromboses. This condition is uncommon in the absence of cirrhosis and other causes of portal hypertension and usually represents a hypercoagulative state [20]. Of 788 CRS/HIPEC procedures at our center, only 3 (0.4%) patients developed this complication. Generally, pregnancy and the postpartum period are associated with an increased risk of venous thrombosis compared to the general population with the highest risk in the postpartum period [21, 22]. Additionally, major surgeries and malignancies also exacerbate this risk [23]. Thus, postpartum women requiring CRS/HIPEC are extremely vulnerable to life-threatening thrombosis. Therefore, adequate antithrombotic prophylaxis is crucial and must be routinely performed during and after CRS/HIPEC in postpartum patients. Physicians should also be cognizant of the high risk for thrombosis in these patients.

## 5. Conclusion

The presented cases demonstrate that LAMN patients with peritoneal carcinomatosis diagnosed during pregnancy may defer definitive treatment of CRS/HIPEC until the postpartum period. Treatment delay allows for spontaneous delivery, breastfeeding, and fertility preservation without compromising the mother or newborn survival. We believe our clinical experience will help guide physicians in clinical decision-making in these challenging situations and reduce both patient and fetus risks.

## Figures and Tables

**Table 1:** Literature review of appendiceal tumors diagnosed during pregnancy.

#	Author (year)	Age	Pathology	PC	Gestational age at dx	Clinical presentation	Treatment during pregnancy	Pregnancy outcome	Staging	Treatment postpregnancy	Time from pregnancy end to treatment	PCI	CC score	Complications	Breast-feeding	Status	Child
*1*	***Present case #1***	*31*	*LAMN*	***Yes***	*2^nd^ Tx (20 weeks)*	*Routine prenatal screening*	*Appy, omentectomy, RSO, peritoneal bx*	*Spontaneous vaginal delivery (38 weeks)*	*MRI abd/pelv at 20 weeks; CT chest/abd/pelv after delivery*	*CRS/HIPEC (Mit-C)*	*4 mos*	*37*	*CC-1*	*POD 15: portal vein thrombosis POD 30: rectal bleeding*	*4 mos+12 mos milk donor*	*5 yrs, NED*	*Healthy*
*2*	*Haase et al. (2010)* [5]	*30*	*Well-differentiated mucinous adenocarcinoma*	***Yes***	*2^nd^ Tx (17 weeks)*	*Routine prenatal screening*	*Appy, RSO, omental bx*	*Early induce vaginal delivery (35 weeks)*	*CT after delivery*	*CRS/HIPEC (Mit-C) + EPIC (5-FU); adjuvant XELOX x8 cycles*	*2.5 weeks*	*28*	*CC-1*	*Neutropenia, prolonged ileus (NGT, TPN), DVT*	*-*	*5 yrs, NED*	*Healthy*
*3*	*Chiverto et al. (2012) [* 6 *]*	*36*	*Infiltrating mucinous adenocarcinoma (T3N1M1a)*	***Yes***	*2^nd^ Tx (18 weeks)*	*Abdominal pain x4 days*	*Dx lap, open appy*	*Terminated with misoprostol*	*Colonoscopy, CT chest/abd/pelv after termination*	*CRS/HIPEC (5-FU + Oxaliplatin; adjuvant FOLFOX x6 Mos*	*-*	*6*	*Complete*	*-*	*NA*	*10 mos, NED*	*NA*
*4*	*Canuto et al. (2016) [* 7 *]*	*38*	*Well-differentiated mucinous adenocarcinoma (pT4apN1)*	***Yes***	*1^st^ Tx*	*Routine prenatal screening (R ovarian mass)*	*Laparoscopic R adnexectomy, peritoneal washing/bx (16 weeks, 4 days)*	*RDS prophylaxis; early C-section (33 weeks, 5 days)*	*-*	*CRS/HIPEC (Mit-C + Cisplatin); adjuvant FOLFOX x8 cycles*	*2 mos*	*-*	*No residual tumor*	*-*	*-*	*2 yrs, NED*	*Healthy*
*5*	*Sebire et al. (2000) [* 8 *]*	*29*	*Moderately differentiated adenocarcinoma; liver metastasis*	***Yes***	*3^rd^ Tx (29 weeks)*	*Abdominal pain, vomiting, UTI*	*None*	*C-section (30 weeks)*	*Abd US (29 weeks); fine-needle liver bx*	*CRS; adjuvant 5-FU/Epirubicin/carboplatin*	*0 days (at C-section)*	*-*	*Residual tumor*	*-*	*-*	*6 mos, AWD*	*Healthy*

6	Donnenfeld et al. (1986) [24]	25	Perforated invasive grade 1 mucinous adenocarcinoma	No	3^rd^ Tx (31 weeks)	Acute appendicitis with peritonitis	Urgent open appy, abscess drain	Induced vaginal delivery (33 weeks)	Chest X-ray, CT abd, colonoscopy 3 days after delivery	R hemicolectomy	9 days	NA	NA	None	-	30 days	Healthy
7	Morgan et al. (2004) [25]	30	Well-differentiated mucinous adenocarcinoma, negative peritoneal washings	No	2^nd^ Tx (26 weeks)	Acute abdomen	Urgent open R hemicolectomy	Spontaneous vaginal delivery (at term)	-	None	NA	NA	NA	None	-	36 mos, NED	-
8	Zeteroğlu et al. (2003) [26]	35	Non-perforated mucinous appendiceal cyctadenocarcinoma	No	2^nd^ Tx (21 weeks)	Symptoms of acute appendicitis	Urgent appy	Terminated with misoprostol (21 weeks)	-	R hemicolectomy, omentectomy	3 days	NA	NA	None	NA	1 yr, NED	NA
9	Casey et al. (2003) [27]	36	Perforated appendiceal mucious cystadenoma	No	2^nd^ Tx (21 weeks)	Symptoms of acute appendicitis	Open appy	-	-	None	NA	NA	NA	-	-	-	-
10	Kalu and Croucher (2004) [28]	42	Mucus adenoma with mucocele	No	1^st^ Tx (5 weeks)	US due to vaginal bleeding	None	Spontaneous miscarriage (anembryonic pregnancy) (7 weeks)	US at 5 weeks (adnexal mass); US in 3 Mos (enlarging ovarian mass)	Laparotomy/appy	3 mos	NA	NA	None	NA	-	NA
11	Idris et al. (2015) [29]	35	Mucocele	No	2^nd^ Tx (22 weeks)	Symptoms of acute appendicitis	Open appy	Spontaneous vaginal delivery (at term)	-	None	NA	NA	NA	None	-	1 yr, NED	-
12	Gilboa et al. (2008) [30]	31	Carcinoid tumor	No	1^st^ Tx (9 weeks)	Symptoms of acute appendicitis	Appy	Spontaneous miscarriage (5 days post-op)	-	-	-	NA	NA	-	-	-	NA
13	Louzi et al.^§^ (2006) [31]	36	Well-differentiated carcinoid tumor	No	3^rd^ Tx (34 weeks)	Symptoms of acute appendicitis	Urgent appy	Vaginal delivery (35 weeks)	-	R hemicolectomy	2 weeks	NA	NA	-	-	23 mos, NED	Healthy
14	Piatek et al. (2016) [32]	28	Well-differentiated carcinoid with gangrenous appendicitis (KI-67: <1%)	No	2^nd^ Tx (25 weeks)	Symptoms of acute appendicitis	Appy	Spontaneous vaginal delivery (38 weeks)	MRI abd at 29 weeks, normal 24-hour urine 5-HIAA; whole body SPECT 3 Mos after delivery	None	NA	NA	NA	None	-	1 yr, NED	Healthy
15	Berrios (1965) [33]	23	Carcinoid tumor	No	1^st^ Tx (2 months)	Symptoms of acute appendicitis	Appy	Spontaneous vaginal delivery (at term)	Negative liver scan during pregnancy	None	NA	NA	NA	-	-	23 mos, NED	Healthy
16	Berrios (1965) [33]	26	Carcinoid tumor	No	1^st^ Tx (10 weeks)	Symptoms of ruptured ectopic pregnancy	Bleeding luteum cyst, incidental appy	-	-	None	NA	NA	NA	-	-	No FU	-
17	Pitiakoudis et al. (2008) [34]	24	Carcinoid tumor (0.5 cm)	No	3^rd^ Tx (32 weeks)	Symptoms of acute appendicitis	Appy	Spontaneous vagina delivery (39 weeks)	-	-	-	NA	NA	None	-	-	Healthy
18	Korkontzelos (2005) [35]	23	Carcinoid tumor (2.2 cm)	No	2^nd^ Tx (16 weeks)	Symptoms of acute appendicitis	Urgent appy	C-section (36 weeks)	-	R hemicolectomy	0 days (at C-section)	NA	NA	-	-	-	Healthy

^§^Article in French. The cases reporting peritoneal spread at presentation are in italics. 5-FU: 5-fluorouracil; 5-HIAA: 5-hydroxyindoleacetic acid; Abd: abdomen; Appy: appendectomy; AWD: alive with disease; Bx: biopsy; CC: completeness of cytoreduction; CRS/HIPEC: cytoreductive surgery with hyperthermic intraperitoneal chemotherapy; C-section: cesarean section; CT: computed tomography; DVT: deep vein thrombosis; Dx: diagnosis/diagnostic; FOLFOX: folinic acid+5-fluorouracil+oxaliplatin; IVF: *in vitro* fertilization; LAMN: low-grade appendiceal mucinous neoplasm; Lap: laparoscopy; Mit-C: mitomycin C; mos: months; NA: not applicable; NED: no evidence of disease; NGT: nasogastric tube; PC: peritoneal carcinomatosis; PCI: peritoneal cancer index; Pelv: pelvis; R: right; RDS: respiratory distress syndrome; SPECT: single-photon emission computed tomography; TPN: total parenteral nutrition; Tx: trimester; US: ultrasound; UTI: urinary tract infection; XELOX: capecitabine+oxaliplatin; Yr (s): year (s).

**Table 2:** Literature review of appendiceal tumors diagnosed during cesarean section.

#	Author (year)	Age	Pathology	PC	Gestational age at dx	Clinical findings during pregnancy	HIPEC referral	C-section surgery	C-section indication	Staging	Postpartum treatment	PCI	CC score	Complications	Breast-feeding	Status	Child
*1*	***Present case #2***	*31*	*LAMN*	***Yes***	*40 weeks*	*Uneventful*	*Yes*	*Appy*	*Premature rapture of membranes*	*CT chest/abd/pelv 3 weeks after C-section*	*CRS/HIPEC (Mit-C) in 3 months*	*27*	*CC-0*	*Prolonged ileus*	*3 mos*	*6 yrs, NED*	*Healthy*
*2*	*Manan et al. (2010)[* 36 *]*	*41*	*Well-differentiated mucinous cystadenocarcinoma*	***Yes***	*At term*	*Uneventful*	*Yes*	*Appy, removal of all gelatinous material*	*Large size of previous babies with complicated delivery*	*Postpartum tumor markers*	*Unknown (referred to outside facility for CRS/HIPEC)*	*-*	*-*	*None*	*-*	*-*	*Healthy*
*3*	*Abdu et al. (2009) [* 37 *]*	*36*	*Well-differentiated mucinous adenocarcinoma*	***Yes***	*40 weeks*	*Lower abdominal pain at 37 weeks*	*Yes*	*R hemicolectomy*	*Failure of cervical dilation*	*-*	*Unknown (referred to outside facility for CRS/HIPEC)*	*-*	*-*	*-*	*-*	*-*	*Healthy*

4	Inubashiri et al. (2019) [38]	24	LAMN	No	38 weeks	Uneventful	NA	Appy	Cephalopelvic disproportion	CT scan 1 month after C-section	None	NA	NA	None	-	6 days	Healthy
5	Yohannes et al. (2019) [39]	31	LAMN	No	38 weeks	Uneventful	NA	Appy	Fetal hypoxia, dysfunctional 2^nd^ stage of labor	CT abd/pelv after C-section	None	NA	NA	-	-	LTFU	Healthy
6	Gallo et al. (2001) [40]	29	Well-differentiated mucinous cystadenocarcinoma	No	38 weeks	Uneventful	NA	Appy	Dysfunctional spontaneous delivery	Postpartum barium enema, abdominal scan, chest X-ray	R hemicolectomy	NA	NA	-	-	5 yrs, NED	Healthy
7	Berrios (1965) [33]	21	Carcinoid tumor	No	At term	Uneventful	NA	Appy	Cephalopelvic disproportion	-	-	NA	NA	-	-	-	Healthy
8	Berrios et al. (1965) [33]	30	Carcinoid tumor	No	38 weeks	-	NA	Appy	-	-	-	NA	NA	-	-	No FU	-
9	Gökaslan et al. (2001) [41]	30	Carcinoid tumor	No	-	-	NA	Appy	-	None	None	NA	NA	-	-	5 mos, NED	Healthy

The cases reporting peritoneal spread at presentation are in italics. Abd: abdomen; Appy: appendectomy; CC: completeness of cytoreduction; CRS/HIPEC: cytoreductive surgery with hyperthermic intraperitoneal chemotherapy; C-section: cesarean section; CT: computed tomography; Dx: diagnosis; FU: follow-up; LAMN: low-grade appendiceal mucinous neoplasm; LTFU: lost to follow-up; Mit-C: mitomycin C; mos: months; NA: not applicable; NED: no evidence of disease; PC: peritoneal carcinomatosis; PCI: peritoneal cancer index; Pelv: pelvis; Yrs: years.

## Data Availability

The data used to support the findings of this study are restricted by the Mercy Medical Center IRB in order to protect patient privacy. Data is available upon request from the corresponding author for researchers who meet the criteria for access to confidential data.
